# 

**Published:** 1910-03-15

**Authors:** 


### ANNOUNCEMENTS.

---

MICHIGAN STATE BOARD OF DENTAL EXAMINERS.—The next regular meeting of the Michigan State Board of Dental Examiners for the examination of applicants for registration will be held at Ann Arbor, beginning Monday, June 20th, and continuing through the 25th. Applications must be in the hands of the secretary at least 14 days before the examination, and should be addressed to

A. W. HAIDLE, *Sec.-Treas.*,
Negaunee, Mich.

---

NEW ORLEANS ODONTOLOGICAL SOCIETY.—At the annual meeting of he Odontological Society of New Orleans the following officers were elected to serve for the ensuing year: President, Dr. J. C. Crimen; Vice-President, Dr. E. L. Fortier; Sec.-Treas., Dr. St. Clair Duke; Executive Com mittee, Drs. L. D. Archinard, H. P. Magruder, and Paul De Verges. Suitable resolutions were passed on the death of John J. Archinard, A.M., M.D., honorary member of this Society. St. Clair Duke, *Secretary.*

---

Canadian Dental Association and Ontario Dental Society.—These Societies will hold a joint session at Toronto, Canada, May 31, June 1, 2 and 3, 1910.

---

Kentucky State Dental Association.—The Forty-first annual meeting of the Kentucky State Dental Association will be held in Louisville, Ky., May 26, 27, 28, 1910.

An unusually interesting and profitable programme is being arranged for this year, and a cordial invitation is extended to all ethical members of the profession.

W. H. Randall, *Secretary.*

---

Illinois State Dental Society.—The 46th annual meeting of the Illinois State Dental Society will be held in Springfield May 17, 18, 19, 20, 1910. In addition to an especially good list of essays and clinics, an unusually attractive supply exhibit is being planned.

J. F. F. Waltz, *Secretary,*
Decatur, Illinois.

---

New Jersey State Board.—The New Jersey State Board of Registration and Examination in Dentistry will hold their semi-annual Examination in the Assembly Chamber of the State House at Trenton, N. J., July 5th, 6th, 7th,

1910. Practical work on the 5th, and the theoretical examination the 6th, and 7th. All applications must be in the hands of the Secretary ten days prior to the meeting. Applicants must send preliminary credentials and photograph with the application or it will not be accepted. Sessions begin promptly at 8 A. M., each day.

CHARLES A. MEEKER, D.D.S.,
29 Fulton St., Newark, N. J.

—✦—

NATIONAL ASSOCIATION OF DENTAL EXAMINERS.—The Twenty-eighth annual session of the National Association of Dental Examiners will hold their meeting in the "New Hotel Savoy", Denver, Colorado, commencing Monday July 25th, at 10 A. M. The rates will be $2.00 per day for one and $3.00 per day for two persons in a room, European plan; large room for one or two with private bath $4.00 and $5.00 per day. Meeting and Committee rooms at the service of the Association, free and every accommodation extended. An early mail reservation is requested, the time being the busy season. A full representation from every state in the United States is earnestly desired.

J. J. WRIGHT, D.D.S., *President*,
Wells Bldg., Milwaukee, Wis.
CHARLES A. MEEKER, D.D.S., *Sect.*,
29 Fulton St., Newark, N. J.

—✦—

NATIONAL DENTAL ASSOCIATION—SOUTHERN BRANCH. To the Officers and Members of the Southern Branch of the National Dental Association.

Gentlemen:

We beg to announce that the date fixed for the coming joint meeting of the Southern Branch and the Texas State

Dental Association has been changed from the date previously announced to May 4th, 5th and 6th, 1910.

This date does not conflict with the time of meeting of any state society within the Southern Branch territory, and we hope for a large attendance.

The holidays being now passed and the date of meeting approaching, it behooves us to make preparation accordingly, if we would have a meeting of a character to do credit to the section we represent, and we trust that each member will exert himself to contribute something toward this end.

Our Texas brothers are giving us their heartiest support and promise us the time of our lives.

Announcement of railroad rates, preliminary program, etc., will follow later.

Let nothing interfere with your participation in the pleasures and profits of this meeting.

Fraternally yours,

J. E. CHACE, *Pres. Sou. Branch.*
W. G. MASON, D.D.S., *Cor. Sect.*

---

ORAL HYGIENE, A NATIONAL CONVENTION, CLEVELAND, OHIO, MARCH 18th, 1910.—President Taft and the Governors of the United States invited to attend the Convention. Believing that the wealth and general prosperity of a nation of people depends upon a condition of healthfulness in both body and mind—that there is not a disease to which the human body is liable that is not aggravated by an unhealthy condition of the mouth—that many diseases are in fact, originally caused by neglected teeth and malnutrition—that health is impaired, beauty marred, happiness destroyed, and life shortened by the deplorable ignorance of the hygienic laws governing the preservation of these important organs; the dentists of this country and other educators have inaugurated what promises to be the greatest campaign ever organized for the abolition of disease. This great humanitarian work will be carried on under the auspices of the Oral Hygiene Committee of the National Dental Association, and will be started in Cleveland, Ohio, where an examination of the school children's mouths by the City Society showed that practically 97 per cent. were in need of attention from a nutritional and hygienic standpoint. The work in Cleveland is only made possible by the hearty support and co-operation of the Cleveland Dental Society and the Ohio State Dental Society. The committee believing that our public schools have served as an incubator for the developing and disseminating of more disease than any one single cause known, and that the most effective way of reaching the present generation of Americans is through their children; have deemed it best to concentrate their attention opon the coming generation.

Through the co-operation of the Board of Education of Cleveland, the committee and the Cleveland Dental Society have established six free Public School Dental Clinics, where school children will be treated for one year at the expense of the committee with the following objects:

1st. To show the general public conditions as they actually exist.

2nd. To show that by proper instruction and care of the mouth that 50 to 75 per cent. of the illnesses among school children will be eliminated.

3rd. That with the mouth and teeth in perfect condition and the food properly cared for while in the mouth, the working efficiency of each pupil will be increased from 50 to a much higher percentage.

4th. To secure data which will cause the states to establish and maintain such inspection and care as will insure the best possible working efficiency in our schools.

5th. To show by comparison of data in schools where the examinations and instruction is given and in those where it is not, that the former are in better condition mentally, morally and physically, than the latter, and to solicit the support of the public in checking and stamping out a condition which has been proven to be fast undermining the life and health of the nation, a condition which if the dental profession were doubled and trebled many times there would not be enough members to handle it.

On the morning of March 18th, 1910, these six free dental clinics will be opened to the public by the members of the National Committee, one committee man formally opening each clinic.

In the afternoon of the same day various members of the committee will be called upon to address a meeting composed of Educational Authorities and School Teachers, not only of Cleveland, but from other cities all over the United States as well. It will be the mission of the afternoon meeting to thoroughly explain the value of each particular part of the work and also the part which they and the public must play in making a success of the same. At this time all phases of the question of public school work will be fairly considered and discussed.

At four-thirty o'clock in the evening the Cleveland Dental Society will give their regular informal banquet and entertain the visiting Dentists and Educators from all over the country.

At eight o'clock the main meeting of the day will be held in Grays Armory, seating some 4000 people. An invitation will be extended to the President of the United States to be present or send a personal representative. The attention of every Governor will be called to the alarming conditions which exist in our public school system, and they will be invited to be present or send representatives. The Educational Authorities of all leading cities of the United States as well as the leading physicians and dentists will be invited to be present, as also will the Cleveland public.

Invitations have been issued to Ex-Pres. Elliott of Harvard University; Mr Horace Fletcher; Dr. W. A. Evans, Commissioner of Health, Chicago; and Dr. Burton Lee Thorpe, President of the National Dental Association, to speak upon this subject in a way that will impress upon every one present the value of perfectly healthy mouths.

Immediately after the inauguration of the campaign in Cleveland, it is the committee's intention to commence this same work in other large cities, especially in Washington, D.C., where possibly more good could be done than in any other city in the country.

To further carry on this work it is the desire of the National Committee to have the president of every state society appoint a committee on Oral Hygiene to work in connection with the National Committee and to have through them, every sub or independent organization in the states elect or appoint committees to work in connection with the National Committee. If this can be done, every organization that is working along the Oral Hygiene line will report to us directly upon what they are accomplishing and when anything new that is worthy of consideration comes from any source whatsoever, it will be compiled along with other good points, printed and mailed to the other committees at work throughout the Country, as well as to school boards, Chambers of Commerce, boards of health, charity institutions and every other organization which may be brought into play to advance the interests of this work.

This will give the movement the benefit of the best minds throughout the country and will send a wave over the land which will do far more good in one year than could be accomplished in years by each individual organization working separately.

Such a system, successfully inaugurated throughout the length and breadth of our continent, would soon make itself evident in a noticeable narrowing of the broad swath annually cut through the columns of our school children by mal-nutrition and disease. Keep pure the mouth, the Gateway of health.—Paul C. White, D.M.D.

